# Supplementary material for: Development and validation of genome-informed and multigene-based qPCR and LAMP assays for accurate detection of Dickeya solani: a critical quarantine pathogen threatening the potato industry
Source: Microbiol Spectr. 2024 Dec 11;13(1):e00784-24. doi: 10.1128/spectrum.00784-24 (PMC11723575; doi:10.1128/spectrum.00784-24)
Supplement: Supplemental figure and table — Fig. S1: Validation of loop-mediated isothermal amplification (LAMP) assay for specific detection of D. solani. Table S1: Bacterial strains used for validation of loop-mediated isothermal amplification (LAMP) and multi-gene-based multiplex TaqMan real-time qPCR. [file spectrum.00784-24-s0001.docx]

**Supplement Materials**

**
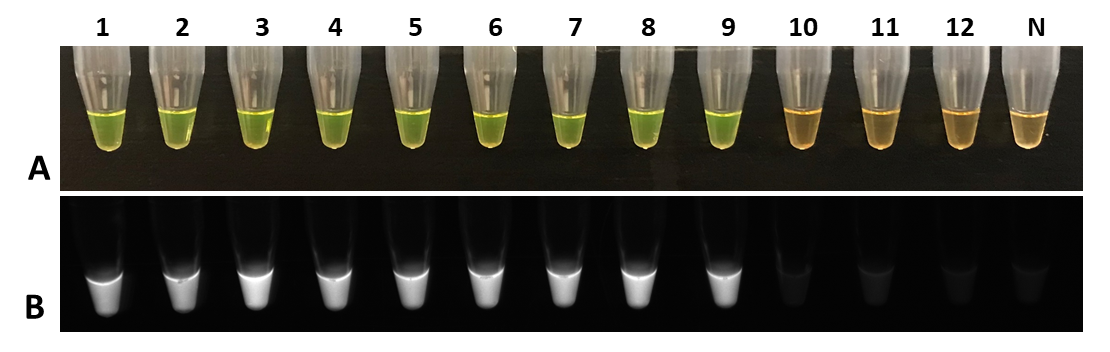
**

**Supplement Figure 1**: Validation of loop mediated isothermal amplification (LAMP) assay for specific detection of *D. solani* from artificially inoculated “disease-free potato” tubers infected plant samples. (A) Visual observation of LAMP results after adding SYBR Green dye in the amplified LAMP products; (B) LAMP results under UV light. Tube 1 is a positive control (genomic DNA of *D. solani* A6295), tubes 2-9 are tubers inoculated with *D. solani* strain A5582, 6289, A6291, A6292, A6295-A6298, tubes 10-11 tuber inoculated with *D. dianthicola* (PL25) and *D. paradisiaca* (A6064), respectively; tube 12 is healthy potato tuber, and tube N is a non-template control (NTC; water).

**Supplement Table 1**: Bacterial strains used for validation of loop mediated isothermal amplification (LAMP) and multi-gene based multiplex TaqMan real-time qPCR for specific detection of *Dickeya solani.*

| Strain | Strain No. | Other ID | Geographic location | Host | Accession no. | LAMP | TaqMan qPCR Mean Ct (SD) | | | | |
| --- | --- | --- | --- | --- | --- | --- | --- | --- | --- | --- | --- |
|  |  |  |  |  |  |  | Yellow (Genus) | Orange  (Dso-P1) | Green  (Dso-P2) | Crimson  (UIC) |  |
| ***Inclusivity Panel*** |  |  |  |  |  |  |  |  |  |  |  |
|  |  |  |  |  |  |  |  |  |  |  |  |
| *Dickeya solani* | PRI 2187 | A5581 | Israel | *Solanum tuberosum* | MH453540 | + | 14.80 (0.03) | 15.92 (0.05) | 15.52 (0.07) | 26.43 (0.22) |  |
| *D. solani* | PRI 2188 | A5582 | Israel | *S. tuberosum* | MH453541 | + | 21.56 (0.04) | 22.35 (0.17) | 22.46 (0.06) | 23.73 (0.09) |  |
| *D. solani* | LMG25615 | A6288 | Belgium | *S. tuberosum* | - | + | 15.78 (0.04) | 16.82 (0.10) | 16.59 (0.06) | 24.79 (0.06) |  |
| *D. solani* | LMG25865 | A6289 | Belgium | *S. tuberosum* | - | + | 14.84 (0.05) | 15.90 (0.02) | 15.59 (0.04) | 24.56 (0.06) |  |
| *D. solani* | LMG25990 | A6291 | Netherlands | *Hyacinthus orientalis* | - | + | 14.37 (0.01) | 15.33 (0.08) | 14.89 (0.03) | 24.45 (0.10) |  |
| *D. solani* | LMG25993 | A6292 | Netherlands | *S. tuberosum* | - | + | 16.63 (0.07) | 17.46 (0.05) | 17.32 (0.08) | 22.46 (0.04) |  |
| *D. solani* | LMG27549 | A6294 | Ireland | *S. tuberosum* | - | + | 14.68 (0.07) | 15.56 (0.14) | 15.34 (0.06) | 23.80 (0.25) |  |
| *D. solani* | LMG27550 | A6295 | Finland | *S. tuberosum* | - | + | 15.12 (0.01) | 16.24 (0.14) | 15.83 (0.01) | 24.59 (0.17) |  |
| *D. solani* | LMG27552 | A6296 | United Kingdom | *S. tuberosum* | - | + | 14.64 (0.03) | 15.70 (0.08) | 15.39 (0.04) | 25.68 (0.03) |  |
| *D. solani* | LMG27553 | A6297 | France | *S. tuberosum* | - | + | 14.71 (0.05) | 15.96 (0.09) | 15.50 (0.07) | 24.34 (0.22) |  |
| *D. solani* | LMG27554 | A6298 | Poland | *S. tuberosum* | - | + | 16.35 (0.06) | 17.33 (0.19) | 17.15 (0.12) | 23.99 (0.08) |  |
| ***Exclusivity Panel*** | | | | | | | | | | | |
| *D. aquatica* | LMG27354 | A6293 | United Kingdom | River water | MN812276 | - | 16.82 (0.03) | - | - | 24.70 (0.10) |  |
| *D. chrysanthemi* | A5415 | CFBP2048 | USA | *Chrysanthemum* | MH453538 | - | 21.53 (0.01) | - | - | 25.17 (0.05) |  |
| *D. chrysanthemi* | A5641 | CFBP 1270 | Denmark | *Parthenium* | MH453539 | - | 33.24 (0.31) | - | - | 25.93 (0.14) |  |
| *D. chrysanthemi* | A5648 | 29-7-1C | Hawaii, USA | Irrigation water | - | - | 16.17 (1.31) | - | - | 22.89 (0.08) |  |
| *D. chrysanthemi* | A5468 | 17-2-3C | Hawaii, USA | Irrigation water | - | - | 17.62 (0.1) | - | - | 23.04 (0.09) |  |
| *D. dadantii* | A6066 | CFBP1889 | Malaysia | *A. comosus* | MK208958 | - | 22.13 (0.19) | - | - | 25.18 (0.07) |  |
| *D. dadantii* | A6067 | CFBP1890 | Malaysia | *A. comosus* | MK208959 | - | 19.88 (0.08) | - | - | 22.99 (0.15) |  |
| *D. dadantii* | A5642 | CFBP 3855 | France | *Saintpaulia* | MH453542 | - | 17.16 (0.03) | - | - | 25.41 (0.13) |  |
| *D. dadantii* | A5416 | CFBP1269 | Comoros, Africa (Comoro Isle) | *Pelargonium capitatum* | MK208944 | - | 17.22 (0.06) | - | - | 25.40 (0.05) |  |
| *D. dadantii* | A5419 | CFBP2051 | USA | *Dieffenbachia* sp. | MK208945 | - | 15.05 (0.04) | - | - | 22.51 (0.03) |  |
| *D. dadantii* | A5643 | CFBP 6467 | Martinique | Musa sp. | MK208950 | - | 19.06 (0.02) | - | - | 25.43 (0.11) |  |
| *D. dadantii* | A5576 | PRI 2120 | Comoros, Africa | *P. capitatum* | MK208948 | - | 17.96 (0.07) | - | - | 24.73 (0.07) |  |
| *D. dadantii* | A1042 | A1042-1 | Hawaii, USA | *Philodendron sp.* | - | - | 11.31 (0.48) | - | - | 23.52 (0.20) |  |
| *D. dadantii* | A1955 | D5 | Missouri, USA | *Zea mays* | - | - | 11.86 (0.28) | - | - | 22.99 (0.10) |  |
| *D. dadantii* | A6060 | CFBP3698 | Cuba | *Musa sp.* | MK208956 | - | 15.65 (0.02) | - | - | 22.22 (0.07) |  |
| *D. dadantii* | A6061 | CFBP1247 | USA | *Dieffenbachia picta* | MK208957 | - | 20.35 (0.02) | - | - | 23.21 (0.05) |  |
| *D. dianthicola* | A5418 | CFBP1200 | UK | *Dianthus caryophyllus* | MK208961 | - | 16.61 (0.15) | - | - | 22.81 (0.05) |  |
| *D. dianthicola* | A5566 | PRI 1363 | The Netherlands | *S. tuberosum* | MK208962 | - | 18.87 (0.05) | - | - | 25.16 0.05) |  |
| *D. dianthicola* | A5567 | PRI 1370 | The Netherlands | *S. tuberosum* | MK208963 | - | 18.28 (0.08) | - | - | 25.22 (0.09) |  |
| *D. dianthicola* | A5568 | PRI 1372-A | The Netherlands | *S. tuberosum* | MK208964 | - | 17.52 (0.03) | - | - | 25.35 (0.12) |  |
| *D. dianthicola* | A5569 | PRI 1372-B | The Netherlands | *S. tuberosum* | MK208965 | - | 15.99 (0.01) | - | - | 24.82 (0.06) |  |
| *D. dianthicola* | A5570 | PRI 1600 | The Netherlands | *S. tuberosum* | MK208966 | Nt | 24.37 (0.16) | - | - | 25.33 (0.07) |  |
| *D. dianthicola* | A5572 | PRI 1741-B | The Netherlands | *S. tuberosum* | MK208946 | - | 19.18 (0.06) | - | - | 27.37 (0.44) |  |
| *D. dianthicola* | A5573 | PRI 2114 | United Kingdom | *Dianthus caryophyllus* | MK208947 | Nt | 15.63 (0.56) | - | - | 25.46 (0.04) |  |
| *D. dianthicola* | A5644 | CFBP2015 | France | *S. tuberosum* | MK208951 | - | 17.31 (0.15) | - | - | 24.53 (0.03) |  |
| *D. dianthicola* | A5645 | CFBP 4155 | The Netherlands | *Kalanchoe blossfeldiana* cv. *maes* | MK208952 | - | 18.61 (0.26) | - | - | 24.82 (0.07) |  |
| *D. dianthicola* | A6058 | CFBP1982 | France | *Dahlia* | MK208953 | - | 18.26 (0.08) | - | - | 24.89 (0.11) |  |
| *D. dianthicola* | A6059 | CFBP3706 | Switzerland | *Cichorium intybus* | MK208955 | - | 19.51 (0.05) | - | - | 24.88 (0.09) |  |
| *D. dianthicola* | PL23 | GBp10B | Hawaii, USA | *S. tuberosum* | MK189270 | - | Nt | Nt | Nt | Nt |  |
| *D. dianthicola* | PL24 | GBp11A | Hawaii, USA | *S. tuberosum* | MK189271 | - | Nt | Nt | Nt | Nt |  |
| *D. dianthicola* | PL25 | GBp21C | Hawaii, USA | *S. tuberosum* | MK189268 | - | 13.08 (0.09) | - | - | 23.73 (0.15) |  |
| *D. fangzhongdai* | CFBP 8607 |  | China | *Pyrus pyrifolia* | - | - | 10.87 (0.39) | - | - | 22.50 (0.07) |  |
| *D. lacustris* | LMG30899 |  | France | Water | - | - | 18.07 (0.20) | - | - | 32.78 (1.5) |  |
| *D. undicola* | LMG30903 |  | Malaysia | Fresh water | - | - | 18.92 (0.40) | - | - | 30.11 (0.2) |  |
| *D. oryzae* | A5307 | 3-6 | Hawaii, USA | *Ananas comosus* | - | - | 13.02 (0.23) | - | - | 22.95 (0.05) |  |
| *D. zeae* | A5376 | 14-1-1A | Hawaii, USA | *A. comosus* | - | - | 12.00 (0.05) | - | - | 22.39 (0.19) |  |
| *D. zeae* | A5423 | CFBP6466 | Martinique | *A. comosus* | MH453536 | - | 19.08 (0.08) | - | - | 25.40 (0.06) |  |
| *D. zeae* | A5511 | 16-12-5N | Hawaii, USA | *A. comosus* | - | - | 14.34 (0.11) | - | - | 22.53 (0.08) |  |
| *D. zeae* | A5623 | 29-5 | Hawaii, USA | *A. comosus* | - | - | 15.75 (0.26) | - | - | 23.13 (0.06) |  |
| *D. zeae* | A5614 | 26-1 | Hawaii, USA | *A. comosus* | - | - | 16.17 (0.13) | - | - | 23.13 (0.22) |  |
| *D. zeae* | A5577 | PRI 2121 | Malaysia | *A. comosus* | MK208949 | - | 19.95 (0.15) | - | - | 25.42 (0.14) |  |
| *D. zeae* | A6056 | 3 leaf | Hawaii, USA | *Ananas comosus* | MH453535 | - | 19.41 (0.17) | - | - | 25.57 (0.04) |  |
| *D. zeae* | A6069 | CFBP1277 | USA | *Z. mays* | MK208960 | - | 20.82 (0.06) | - | - | 25.82 (0.10) |  |
| *D. zeae* | PL47 |  | Hawaii, USA | *Brassica oleracea var. sabellica* | - | - | 22.56 (0.07) | - | - | 25.89 (0.11) |  |
| *D. zeae* | A5265 | 1-3A | Hawaii, USA | *Ananas comosus* | MK189273 | - | Nt | Nt | Nt | Nt |  |
| *D. paradisiaca** | A5420 | CFBP4178 | Colombia | *Musa paradisiaca* | MK208942 | - | 18.30 (0.06) | - | - | 25.46 (0.06) |  |
| *D. paradisiaca** | A5579 | PRI2127 | Colombia | *M. paradisiaca* | MK208943 | - | 17.80 (0.09) | - | - | 25.63 (0.08) |  |
| *D. paradisiaca** | A5688 | CFBP 3699 | Cuba | *Zea mays* | - | - | 12.12 (0.08) | - | - | 23.54 (0.10) |  |
| *D. paradisiaca** | A6064 | CFBP 3696 | Cuba | *Musa sp.* | - | - | 11.85 (0.15) | - | - | 23.21 (0.11) |  |
| *Rathayibacter rathayi* | A1152 | ATCC 13659 | UK | Grass | MH605382 | - | - | - | - | 23.50 (0.08) |  |
| *R. rathayi* | LMG3717 | ATCC13659 | United Kingdom | *Dactylis glomerata* | - | - | - | - | - | 23.29 (0.14) |  |
| *R. iranicus* | LMG3677 | CFBP807 | *Triticum aestivum* | Iran | - | - | - | - | - | 23.28 (0.12) |  |
| *R. tritici* | LMG3726 | ICMP 2624 | *Triticum aestivum* | Egypt | - | - | - | - | - | 23.32 (0.12) |  |
| *R. toxicus* | CS29 |  |  |  | - | - | - | - | - | 25.78 (0.05) |  |
| *Pectobacterium carotovorum* | A5278 | 1-#21 | Hawaii, USA | Irrigation water | MH453511 | - | - | - | - | 25.38 (0.08) |  |
| *P. carotovorum* | A6273 | BA17 | Hawaii, USA | *Solanum lycopersicum* | MK453527 | - | - | - | - | 26.18 (0.44) |  |
| *P. carotovorum* | A5280 | 1-#31 | Hawaii, USA | Irrigation water | MH453512 | - | - | - | - | 25.86 (0.13) |  |
| *P. carotovorum* | A5368 | 5X | Hawaii, USA | *Aglaonema* sp. | MH453510 | Nt | - | - | - | 25.09 (0.30) |  |
| *P. carotovorum* | A5350 | 5C | Hawaii, USA | *Aglaonema* sp. | MK208940 | - | - | - | - | 25.74 (0.10) |  |
| *P. carotovorum* | A5352 | T-15 | Hawaii, USA | *Aglaonema sp.* | MH453529 | - | Nt | Nt | Nt | Nt |  |
| *P. odoriferum* | A1089 | QR-11 | California, USA | *Capsicum annuum* | MH453518 | - | - | - | - | 25.12 (0.51) |  |
| *P. carotovorum* | PL73 | 51C | Hawaii, USA |  |  | - | - | - | - | 24.90 (0.28) |  |
| *P. odoriferum* | A2686 | E43 | Hawaii, USA | *Brassica oleraceae* var. *capitata* | MH453519 | - | Nt | Nt | Nt | Nt |  |
| *P. betavasculorum* | A6165 | Ecb1 | California, USA | *B. vulgaris* | MK250993 | - | - | - | - | 25.49 (0.31) |  |
| *P. betavasculorum* | A6167 | Ecb6 | California, USA | *B. vulgaris* | MK250994 | - | Nt | Nt | Nt | Nt |  |
| *P. wasabiae* | LMG8444 |  | Nagano Prefecture Japan | *Eutrema wasabi* | - | - | - | - | - | 33.14 |  |
| *P. brasiliense* | A6152 | WPP165 | Wisconsin, USA | *S. tuberosum* | MH453521 | Nt | - | - | - | 25.36 (0.20) |  |
| *P. brasiliense* | A6149 | WPP5 | Wisconsin, USA | *S. tuberosum* | MH453522 | - | Nt | Nt | Nt | Nt |  |
| *P. brasiliense* | PL108 | PS60E | Hawaii, USA | *S. tuberosum* | MN428429 | - | - | - | - | 24.69 (0.39) |  |
| *P. aroidearum* | A5348 | 2d | Hawaii, USA | *Aglaonema sp.* | MK182847 | - | - | - | - | 25.67 (0.16) |  |
| *P. aroidearum* | LMG2417 | A6281 | South Africa | *Zantedeschia aethiopica* | MN990003 | - | - | - | - | 25.63 (0.34) |  |
| *P. parmentieri* | PL30 | GBp2-1 | Hawaii, USA | *S. tuberosum* | MN428432 | Nt | - | - | - | 25.06 (0.09) |  |
| *P. parmentieri* | PL32 | GBp4-3 | Hawaii, USA | *S. tuberosum* | MN428433 | Nt | - | - | - | 25.09 (0.27) |  |
| *P. parmentieri* | A1852 | M784 | Colorado, USA | *S. tuberosum* | MH453534 | - | Nt | Nt | Nt | Nt |  |
| *P. peruviense* | LMG30269 |  | Peru | *S. tuberosum* | - | - | - | - | - | 25.79 (0.21) |  |
| *P. atrosepticum* | LMG2375 |  | United Kingdom | *S. tuberosum* | - | - | - | - | - | 25.79 (0.33) |  |
| *P. atrosepticum* | A1850 | IPM 1260 | Colorado, USA | *Colorado, USA* | MH453513 | - | Nt | Nt | Nt | Nt |  |
| *P. betavasculorum* | LMG2461 |  | United States | *Beta vulgaris* | - | - | - | - | - | 25.91 (0.17) |  |
| *P. polaris* | PL63 | K-G | Hawaii, USA | *Brassica oleraceae* | MK189265 | - | Nt | Nt | Nt | Nt |  |
| *P. polaris* | ICMP9180 |  | Netherlands | *S. tuberosum* | - | - | - | - | - | 29.73 (1.5) |  |
| *P. versatiles* | ICMP 9168 |  | Netherlands | *S. tuberosum* | - | - | - | - | - | 30.37 (1.6) |  |
| *P. versatiles* | PL62 | Pot 1 | Hawaii, USA | *S. tuberosum* | - | - | - | - | - | 25.12 (0.09) |  |
| *P. versatiles* | A1838 | UC 202.1B | California, USA | *S. tuberosum* | - | - | - | - | - | 25.40 (0.16) |  |
| *P. punjabense* | LMG30622 |  | Pakistan | *S. tuberosum* | - | - | - | - | - | 33.67 (0.2) |  |
| *P. actinidiae* | LMG26004 |  | South Korea | *Actinidia chinensis* | - | - | Nt | Nt | Nt | Nt |  |
| *P. cacticida* | LMG 2720 | A6283 | Arizona, USA | *Carnegiea gigantea* | MN990005 | - | Nt | Nt | Nt | Nt |  |
| *P. cypripedii* | A5186 | ATCC29267 | California, USA | *Cypripidium sp.* | MK182846 | - | Nt | Nt | Nt | Nt |  |
| *Pantoea* sp. | A1865 | YP-1 purple | Hawaii, USA |  | MK182843 | - | - | - | - | 26.02 (0.40) |  |
| *Pantoea* sp. | A1867 | F2 c. papaya-purple | Hawaii, USA | *C. papaya* | MK182844 | - | - | - | - | 26.31 (0.32) |  |
| *Pantoea* sp. | A1869 | F7 | Hawaii, USA |  | MK182845 | - | Nt | Nt | Nt | Nt |  |
| *Pantoea* sp*.* | A5358 | J9 | Hawaii, USA | *Carica papaya* | MK182848 | - | - | - | - | 25.95 (0.08) |  |
| *Pantoea agglomerans* | A6222 | DP138 | Wisconsin, USA | *Z. mays* | MH547382 | Nt | - | - | - | 25.36 (0.34) |  |
| *P. agglomerans* | A5513 |  | Hawaii, USA | Ornamental | MK182849 | - | Nt | Nt | Nt | Nt |  |
| *P. cypripedii* | LMG1268 |  | United States | *Cyprepedium sp.* |  | - | - | - | - | 26.19 (0.31) |  |
| *Enterobacter cloacae* | A5149 | B193 | Hawaii, USA | *Zingiber officinale* | MK182850 | - | - | - | - | 25.94 (0.03) |  |
| *Klebsiella aerogenes* | A3131 | ATCC13048 | - | - | MK208954 | - | - | - | - | 25.78 (0.41) |  |
| *Klebsiella* sp*.* | A6223 | DP140 | Iowa, USA | *Z. mays* | MH547379 | - | - | - | - | 26.02 (0.16) |  |
| *Erwinia amylovora* | A1084 | QR-6 |  | *Pyrus* sp. | MK182851 | - | - | - | - | 25.75 (0.13) |  |
| *Erwinia* sp. | A5367 | 4C | Hawaii, USA | *Aglaonema* sp. | MK243480 | - | Nt | Nt | Nt | Nt |  |
| *Ralstonia pseudosolanacearum* | A6117 | S-6 | Guam, USA | *Casuarina equisetifolia* | - | Nt | - | - | - | 26.21 (0.23) |  |
| *R. solanacearum* | A5685 |  | Florida, USA | *Solanum lycopersicum* | - | Nt | - | - | - | 25.79 (0.13) |  |
| *R. solanacearum* | A3450 | 30 | Trinidad | *Solanum lycopersicum* | MK243481 | - | Nt | Nt | Nt | Nt |  |
| *X. paseoli* pv. *dieffenbachiae* | PL36 |  | Hawaii, USA | *Anthurium* sp. | - | - | - | - | - | 25.60 (0.14) |  |
| *X. phaseoli* pv. *dieffenbachiae* | D182 |  | Hawaii, USA | *Anthurium* sp. | - | - | - | - | - | 25.46 (0.31) |  |
| *Clavibacter michiganensis* | A2058 | H-160 | Idaho, USA | *S. lycopersicum* | MH560477 | - | - | - | - | 26.18 (0.58) |  |
| *C. michiganensis* | A2645 | S47 | California, USA | *S. lycopersicum* | MH560480 | Nt | - | - | - | 25.22 (0.30) |  |
| *C. michiganensis* | A4758 | N 212 | China | *S. lycopersicum* | MH560484 | - | Nt | Nt | Nt | Nt |  |
| *C. michiganensis* | A4690 | CMM 461 | Portugal | *S. lycopersicum* | MH560483 | - | Nt | Nt | Nt | Nt |  |
| *C. insidiosus* | A1149 | ATCC 10253 | Kansas, USA | *Medicago sativa* | MH560501 | - | - | - | - | 26.37 (0.19) |  |
| *C. chiloensis* | A6101 | ZUM3936 &  ATCC BAA-2690 | Chile, USA | *S. lycopersicum* | MH560495 | Nt | - | - | - | 26.26 (0.53) |  |
| *C. tessellarius* | A6109 | ATCC 33566 | Nebraska, USA | *Triticum aestivum* | MH560502 | Nt | - | - | - | 26.07 (0.66) |  |
| *C. californiensis* | A6134 | C55 | California, USA | *S. lycopersicum* | MH560499 | Nt | - | - | - | 26.01 (0.38) |  |
| *C. phaseoli* | A6135 | LPPA982 | Spain | *Phaseolus vulgaris* | MH560500 | Nt | - | - | - | 25.96 (0.22) |  |
| *C. capsici* | A6113 | 1647, P5006 | China | *C. annuum* | MH560497 | Nt | - | - | - | 26.27 (0.19) |  |
| *C. sepedonicus* | A2041 | R8 | Denmark | *S. tuberosum* | MK560493 | - | - | - | - | 25.94 (0.23) |  |
| *C. sepedonicus* | A6172 | ATCC33113 | Canada | *S. tuberosum* | MK560494 | - | - | - | - | 25.85 (0.10) |  |
| *C. nebraskensis* | A6211 | DP139B | Texas, USA | *Z. mays* | MH560469 | - | - | - | - | 25.70 (0.18) |  |
| *C. nebraskensis* | A6206 | DP117 A | Nebraska, USA | *Z. mays* | MH560464 | - | Nt | Nt | Nt | Nt |  |
| *C. nebraskensis* | A6094 | NCPPB2579 | Nebraska, USA | *Z. mays* | MH560472 | - | Nt | Nt | Nt | Nt |  |
| *Curtobacterium flaccumfaciens* | A6266 | 70002^a^ | unknown | *E. pulcherrima* | MH605376 | - | - | - | - | 25.85 (0.07) |  |
| *C. flaccumfaciens^c^* | A6267 | 70008^a^ | unknown | *Tulipa* | MH605377 | - | - | - | - | 25.73 (0.33) |  |
| *C. flaccumfaciens* pv. *pointsettiae* | A1147 | ATCC 9682^T^ | USA | *Euphorbia pulcherrima* | MH605380 | - | Nt | Nt | Nt | Nt |  |
| *Microbacterium* | A6214 | DP101 | Iowa, USA | *Z. mays* | MH547375 | - | - | - | - | 26.22 (0.12) |  |
| *Rhodococcus fascians* | A1151 | ATCC 12975 | USA | Unknown | MH605375 | - | - | - | - | 26.46 (0.19) |  |
| *Bacillus* sp. | A6181 | CC97 | Unknown | Unknown | MK202803 | - | Nt | Nt | Nt | Nt |  |
| *Pseudonomonas psychrotolerans* |  | 5 | Hawaii, USA | *Sorghum halepense* | - | - | - | - | - | 25.51 (0.07) |  |
| *Curtobacterium* sp. |  | 9A | Hawaii, USA | *Panicum maximum* | - | - | - | - | - | 25.37 (0.08) |  |
| *P. stewartii* |  | 13A | Hawaii, USA | *Setaria verticillata* | - | - | - | - | - | 25.37 (0.06) |  |
| *Pseudomonas oryzihabitans* |  | 13B | Hawaii, USA | *Setaria verticillata* | - | - | - | - | - | 25.35 (0.22) |  |
| *Sphingomonas* sp. |  | 39C | Hawaii, USA | *Panicum maximum* | - | - | - | - | - | 25.38 (0.25) |  |
| *X. translucens* |  | 45A | Hawaii, USA | *P. maximum* | - | - | - | - | - | 25.26 (0.15) |  |
| *Rhodococcus* sp. |  | 50A | Hawaii, USA | *Pennisetum setaceum* | - | - | - | - | - | 24.97 (0.43) |  |
| *Agrobacterium* sp. | A2961 | C58 | Newyork, USA | *Prunus avium* | - | - | - | - | - | 25.82 (0.32) |  |
| ***Other species isolated from infected S. tuberosum*** | | | | | | | | | | | |
| *Pseudomonas* sp. | PL172 |  | Hawaii, USA | *S. tuberosum* | - | - | - | - | - | 33.87 ((0.2) |  |
| *Pseudomonas* sp. | PL176 |  | Hawaii, USA | *S. tuberosum* | - | - | - | - | - | 31.92 (1.19) |  |
| *Acinetobacter* sp. | PL175 |  | Hawaii, USA | *S. tuberosum* | - | - | Nt | Nt | Nt | Nt |  |
| *Raoultella* sp. | PL177 |  | Hawaii, USA | *S. tuberosum* | - | - | Nt | Nt | Nt | Nt |  |
| *Delfia* sp. | PL178 |  | Hawaii, USA | *S. tuberosum* | - | - | Nt | Nt | Nt | Nt |  |
| *Soil infected with P. parmentieri* |  |  |  |  |  | - | - | - | - | 30.59 (1.9) |  |
| Healthy *S. tuberosum* |  |  |  |  | - | - | - | - | - | 25.95 (0.05) |  |
| Healthy Soil |  |  |  |  |  | - | - | - | - | 32.93 (2.2) |  |

- Indicates no amplification; + indicates amplification; DSO-P1, DSO-P2- shows amplification for *D. solani*; Nt- not tested; UIC: Universal Internal Control. ±Ct (threshold cycle) values are an average of three replicates. SD: standard deviation. *Newly proposed as genus *Musicola paradisiaca.*
